# Silica: Its Curative Action in the Treatment of Alveolar Abscess

**Published:** 1892-09

**Authors:** Charles H. Taft


### SILICA: ITS CURATIVE ACTION IN THE TREATMENT OF ALVEOLAR ABSCESS.[1]

BY CHARLES H. TAFT, A.B., D.M.D.

THE subject which I wish to present for your consideration is, in my opinion, an important one ; not because I consider the remedy by any means a specific for the general treatment or cure of alveolar abscesses, but because I consider it to be a remedy which, when used with intelligence, will be found to be as useful in the hands of the dentist as it is in those of the physician.

There are probably few among us who do not frequently have cases in practice that seem to call for systemic rather than for surgical or mechanical treatment. When, for instance, pathological conditions are brought about by a disturbance of the vital force, resulting in an abnormal functional activity of an organ or tissue, and when we are called upon to prove to the patient that we have both the knowledge and ability to correct and to overcome such disturbances and thereby to disprove the all too prevalent idea in the public mind of to-day that dentistry is even yet but little better than a tooth-pulling, or even a tooth-saving, art.

Common among the every-day cases coming to us for treatment are alveolar abscesses, the etiology of which we are, of course, familiar with, and concerning which nothing need be said. They are commonly divided into two classes,—the acute and the chronic

---

[1] Read before the American Academy of Dental Science, Boston, April 6, 1892.

forms; the former being such as easily yield to treatment, the latter being such as do not so readily yield. It is this latter class that I wish particularly to consider, and to show how far the suppurative process can be materially modified and controlled, if not, indeed, made to disappear entirely.

To illustrate, permit me to cite a case in practice which I can safely assert has been the most obstinate, with one exception, in yielding to treatment that I have ever had to deal with.

On February 23, 1891, Miss B. presented herself at my office complaining of severe pain in the right inferior second bicuspid, a large corono-mesial filling having been inserted a year previous by a dentist abroad. Upon examination the pulp of the tooth was found to be dead. It was then thoroughly removed and a dressing applied, the cavity being temporarily sealed with gutta-percha. She presented herself the following day with her face badly swollen, and the process of suppuration had advanced to the stage which made the lancing of the abscess a simple and easy matter. Both gutta-percha and cotton dressing were then removed and the cavity allowed to remain open for a few days until the swelling had entirely subsided and a fistulous opening had become established, which appeared, rather curiously, on the hard ridge of the alveolus directly behind the tooth (the sixth-year molar having been previously removed), rather than opposite the extremity of the root, where we should naturally expect to find it.

At the following sitting all traces of pulp-tissue and débris were removed from pulp-chamber and root-canal, and a cotton dressing dipped in creolin applied and temporarily sealed in place.

At the next sitting the dressing was found to be absolutely clean and sweet, and the fistulous opening, instead of healing up as was to be expected,—the exciting cause having been removed,—grew larger, the tissue directly around the opening assumed an angry bluish-red appearance, and the discharge of pus was sufficiently large to be at all times noticeable and very annoying to the patient. A large-sized probe could be easily carried along the fistulous tract to the apex of the root.

Renewed efforts were made to effect a disappearance of the abscess by treatment directly through the root-canal and the fistula itself.

Resort was had to a method which I have on previous occasions found very satisfactory in obstinate cases,—namely, that of passing a very sharp burr along the fistulous tract and cutting away the sac attachment so far as possible from the apex of the root, afterwards syringing it out with a strong solution of peroxide of hydrogen, followed by a weak solution of carbolized water.

The patient presented herself frequently from February 23 to October 3. Upon each occasion the dressing previously applied was found to be without the slightest trace of odor other than its own, while the fistulous opening remained the same, with the same ugly, inflamed appearance and copious discharge of pus.

Both patient and operator were beginning to get somewhat tired of the slow process of healing, and at last I frankly confessed that I had exhausted all the known dental means and methods with which I was conversant for the treatment of alveolar abscess, and remarked that I thought it was now time to see what medicine would do in the case. The tooth had, in the mean time, become much discolored, but, aside from the annoying discharge of pus, was of no discomfort to the patient.

With a knowledge of the physiological and medicinal action of silica, and its remarkable control over the suppurative process, the patient was given six powders in the two-hundredth potency of the drug, with instructions to take one powder dry on the tongue every night before retiring, and at the end of a week to come in and report. At the expiration of a week she presented herself, and there was visible a very decided improvement in the appearance of both fistula and the parts around it, as well as a very marked decrease in the discharge of purulent matter.

The patient remarked that the improvement began to be immediately noticeable the day following the taking of the first powder. The same medicine was then repeated, with instructions to take as before, and at the end of a fortnight to come in again and report.

The next visit showed a continued improvement. The inflammation was slowly but steadily subsiding, likewise the flow of pus, and it required a much smaller-sized probe to be passed along the tract. No further medicine was given, but the patient was instructed to report at the end of another week.

The following visit showed still further improvement, and at first sight the fistula had apparently entirely healed, much to my intense gratification. It had by this time become so small that without knowing its exact location its existence, to a casual observer, would have been not easily discernible, but upon applying pressure upon the ridge a small discharge was seen to emanate from the opening, and a fine probe could still be passed along the entire tract to the apex of the root.

At this stage of treatment the silica was dropped for the time being and causticum given, and following that, a week later, fluoric acid; these also in the two-hundredth dynamization.

No perceptible change or further improvement was visible from the effect of either of these drugs, and the patient reported that the medicine had been without apparent effect, unless it was to continue the improvement which the first medicine had effected. She reported further that the discharge had been so slight as to be noticeable only upon pressure.

Feeling confident that the first selection of the medicine given had been the right one, there being an absolute lack of contingent symptoms to point to any other drug as being the similimum of their totality, and of which there is generally a greater or less number when the case is one that calls for systemic treatment; and knowing also that the curative power of a medicine when homœopathic to the case is greatly increased in proportion to the reduction of the dose to that degree of minuteness at which it will exert a *gentle* curative influence, I again went back to the silica, this time giving it in a much higher potency—the one-hundred-thousandth—and in the divided dose; that is to say, dissolved in water. The patient was instructed to report in a week, but three or four elapsed before she presented herself. The medicine having done its work so well, she had hardly thought it necessary.

This time, to my great satisfaction, as well as to the patient's, the discharge was found to have entirely ceased. The tissues had completely reassumed their normal color and appearance, but the tract still remained; so minute, however, that pressure failed to cause the appearance or further discharge of offensive matter. No further medicine was given and the patient was dismissed.

On March 8 the patient called to inquire if the tooth could not be permanently filled, not the slightest discharge having been noticed since her last visit, two months previous. The tract still remains, very minute as before, but no discharge can be made to come from it in any way, shape, or manner.

What, then, has been accomplished? In the first place, there was a localized disturbance or change in the vital force, brought about by a specific cause, resulting in a perverted nutrition and abnormal functional activity of both the organ itself and the tissues in relation thereto.

Secondly, all surgical efforts having failed to bring about a restoration to health of the parts concerned, and the case then seeming to be one pre-eminently calling for systemic treatment, a remedy was selected whose value and power in controlling the suppurative process is well known to those who practise medicine in accordance with the teachings of Hahnemann, and which, in cases of simple abscess, has a brilliant clinical record.

Thirdly, in this, as in the process of any homœopathic cure, to quote the words of Hahnemann, "by administering a medicinal potency chosen exactly in accordance with the similitude of symptoms, a somewhat stronger similar artificial morbid affection is implanted upon the vital power deranged by a natural disease. This artificial affection is substituted, as it were, for the weaker similar natural disease (morbid excitation), against which the instinctive vital force, now only excited to stronger effort by the drug affection, needs only to direct its increased energy; but, owing to its brief duration, it will soon be overcome by the vital force, which, liberated first from the natural disease, and finally from the substituted artificial (drug) affection, now again finds itself enabled to continue the life of the organism in health."

From what I have stated the impression may go forth that equal success in the treatment of alveolar abscesses might ensue from the indiscriminate employment of this drug as a sort of specific in all forms of this pathological condition, but this is by no means the case.

It would be both interesting and profitable, were it within the limits of the paper, to consider the action of other medicines equally valuable in the treatment of abscesses, and to show why a remedy would prove effectual in one case and totally ineffectual in another; but for the proper selection of any remedy in a given case the fact should not be lost sight of that a knowledge of the physiological or toxical action of a drug is, in the first place, most essential. By this I mean we must know what pathological conditions are brought about when a proving of the drug is made upon the healthy human organism: what tissues are affected, and in what way they are affected; for it is only by comparing the symptoms of your patient with those which different drugs produce upon the healthy body that a correct or scientific knowledge of their curative action can be obtained. Without this knowledge one would wander blindly in making his selection of a remedy.

To some of those who may be unfamiliar with the great number and variety of medicines to be found in the homœopathic materia medica, it may appear incredible or absurd that a substance which in its native form is simply the pure silica of quartz-crystal, and which in its crude state is but an inert mineral substance, can have any medicinal action; and not less incredible and absurd when administered in the potencies I have described. But it is only after such a substance has been triturated with a non-medicinal substance, a process whereby its molecules become comminuted or broken up, that its medicinal properties are evolved. The different potencies of the drug being then carried up by the fluid process or succession, the higher the potency the more powerful its action, and the greater care and discretion in consequence to be exercised in their use.

The action of the drug we are considering is a slow and deep one,—the nutrition of the tissues which come within its sphere of action rather than their functional activity being especially influenced by it, and by reason of its slow action is suited to chronic rather than acute affections. But it is in cases involving profuse suppuration that its value as a therapeutic agent is especially appreciated, where abscesses can be made quickly to come to maturity and the secretion of pus either modified or brought entirely under control.

I have heard it stated by men prominent in our profession that medicine is not and never can be an *exact* science. To this broad statement I feel compelled, through convictions established within me based upon evidence and experience, to take exceptions.

It is not my purpose, however, to encourage discussions of a general character at this time upon what properly belongs to another subject, and upon which much has heretofore been written and said; but speaking for myself alone, and as a dentist who believes most assuredly that medicine (or if I may be allowed to qualify the word by substituting homœopathy) *is* an *exact* science, not a speculative theory; a principle, a law of nature as universal in its application as any of the natural laws which govern the universe; and that dentistry is, or, at least, should be, in a broad sense, a specialty *of* medicine. I cannot conclude my remarks upon the subject before us better than to express the satisfaction I daily find in applying a rational and intelligent knowledge of the materia medica, so far as I understand it, in the treatment of such organic or functional disturbances as properly come within our province as dentists; and where the use of the ordinary and rather limited number of dental therapeutic agents at our command prove either only temporarily useful and satisfactory, or, what is often found in the long run to be the case, wholly unsatisfactory and therefore largely useless.

